# Post-injury and resolution response to repetitive inhalation exposure to agricultural organic dust in mice

**DOI:** 10.3390/safety3010010

**Published:** 2017-02-21

**Authors:** Kristi J. Warren, Todd A. Wyatt, Debra J. Romberger, Isaak Ailts, William W. West, Amy J. Nelson, Tara M. Nordgren, Elizabeth Staab, Art J. Heires, Jill A. Poole

**Affiliations:** 1 Pulmonary, Critical Care, Sleep & Allergy Division; Department of Internal Medicine, University of Nebraska Medical Center (UNMC), 985990 Nebraska Medical Center, Omaha, NE 68198-5990, USA; 2 Department of Environmental, Agricultural, and Occupational Health, University of Nebraska Medical Center, 985990 Nebraska Medical Center, Omaha, NE 68198-5990, USA; 3 Veterans Affairs Nebraska-Western Iowa Health Care System, 4101 Woolworth St., Omaha, NE 68105, USA; 4 Department of Pathology and Microbiology, UNMC, 983135 Nebraska Medical Center, Omaha, NE 68198-3135, USA

**Keywords:** Lung, repair, inflammation, resolution, lymphocyte, agriculture, farm, neutrophil, immunity, swine barn

## Abstract

Inhalation of organic dusts in agricultural environments causes airway inflammatory diseases. Despite advances in understanding the airway response to dust-induced inflammation, less is known about the transition from lung injury to repair and recovery. The objective of this study was to define the post-inflammation homeostasis events following organic dust-induced lung injury. Using an established protocol, mice were intranasally treated with swine confinement facility organic dust extract (ODE) daily for 3 weeks (repetitive exposure) or treated daily with ODE for 3 weeks followed by no treatment for 1–4 weeks (recovery period) whereupon lavage fluid, lung tissue, and sera were processed. During recovery period, a significant decrease was observed in ODE-induced neutrophil levels after 1 week, lymphocytes at 2 weeks, and macrophages at 4 weeks in the lavage fluid. ODE-induced lung cellular aggregates and bronchiolar compartment inflammation were diminished, but persisted for 4 weeks post-injury. Alveolar inflammation resolved at 3 weeks. ODE-induced lung neutrophils were cleared by 3 weeks, B-cells by 2 weeks, and CD3^+^CD4^+^ and CD3^+^CD8^+^ T cells by 4 week recovery period. Collectively, these results identify important processes during recovery period following agricultural dust-induced inflammation, and present possible strategies for improving lung repair and resolution.

## 1. Introduction

Inhalation of agricultural organic dusts cause injury and inflammation in the lungs of exposed workers [1,2]. These organic dust exposures contribute significantly to the development of chronic pulmonary conditions, such as bronchitis and chronic obstructive pulmonary disease (COPD) [3–5]. Exposure to organic dusts results in airway neutrophil and lymphocyte influx, release of inflammatory mediators, and lung parenchymal injury [1,5,6]. The composition of agricultural dust is complex, but is known to contain gram positive and gram negative microbial cell wall components, proteases, and particulate matter [3,7,8]. This complex organic dust activates several lung innate immune pathways that otherwise may not be activated in single agent (e.g. endotoxin, bleomycin) exposure injury models [6,9]. Progress has been made in understanding the mechanisms underlying the inhalant response to acute and repetitive agricultural organic dust/dust extract exposures. Specifically, scavenger receptor A/CD204, Toll-like receptor 2 (TLR2), TLR4, TLR9, and the common adaptor protein, myeloid differentiation factor 88 (MyD88)-dependent pathways have been shown to be important in mediating acute and repetitive swine confinement facility organic dust extract exposure-induced airway inflammatory outcomes in rodent models [10–13]. Furthermore, TLR2 and TLR4 gene polymorphisms have been implicated in modulating lung disease in swine workers [14,15].

The magnitude of the innate immune response and subsequent inflammation is substantial, and if left unresolved can lead to progressive lung function loss over time. In agriculture workers who develop Farmer’s lung disease (hypersensitivity pneumonitis) or COPD [16–18], current management has been focused on improving symptoms and quality of life because there are a lack of treatments to fully restore lung function [19]. While there are several animal models utilized to study environmental exposure-induced lung injury and chronic lung inflammation [9,20], few studies have examined the recovery phase by which lung inflammation and injury resolves following organic dust extract exposures [12]. In this later study [12], up to one week post-exposure was investigated as well as demonstrating an important role for scavenger receptor A signaling. Investigation of further time points has not been examined to the best of our knowledge, but this information could provide insight into potential strategies to augment recovery events following inhalation of inflammatory environmental or occupational aerosols, particularly agricultural organic dust exposures. It is generally recognized that clearance of inciting insult(s) and removal of inflammatory cells is required for establishment of tissue homeostasis [21]. It is likely that a concerted immune response is necessary to resolve agricultural organic dust in the lungs and mediate repair and resolution of tissue damage following such an insult. The objective of this study was to define the post-inflammatory lung resolution events in a time-dependent manner following repetitive swine confinement facility organic dust extract (ODE) exposure.

Using a well-established animal model [6], C57BL/6 mice were repetitively exposed to ODE daily over a 3-week period, and upon cessation of ODE exposure, subsets of mice were euthanized at 1, 2, 3, and 4 weeks after exposure with experimental endpoints quantified. Studies revealed that neutrophil, macrophage, and lymphocyte infiltration persisted up to 3–4 weeks after repetitive ODE exposure ceased. In addition, we further characterized the resolution of lung cellular aggregates in ODE exposed mice by showing that apoptotic events increased during resolution. Amphiregulin, a repair mediator, increased during the resolution phase, and systemic immunoglobulin (Ig) responses remained elevated up to 4 weeks post-injury.

## 2. Methods

### 2.1. Organic dust extract

Aqueous ODE was prepared as previously described [6]. Settled dust was collected from horizontal surfaces (~1 meter above floor level) of swine confinement feeding operations located in Colfax County, Nebraska (population density approximates 25 people per square mile) that housed approximately 400–600 animals with permission granted from owners. Dust (1 g) was incubated in sterile Hank’s Balanced Salt Solution (10 mL; Sigma, St. Louis, MO) at room temperature for 1 hour and centrifuged for 60 min at 2000 × *g*. The final supernate was filter sterilized (0.22 μm), a process that also removes both coarse and fine particles. Endotoxin concentrations in 100% ODE ranged from 1240–1400 EU/mL as determined using the limulus amebocyte lysate assay (Sigma). Muramic acid levels were previously determined by mass spectrometry to be approximately 70 ng/mg [22]; muramic acid is a molecular component of bacterial cell wall peptidoglycans. Stock ODE was batched prepared, stored at −20°C, and aliquots were diluted for each experiment to a final concentration (vol/vol) of 12.5% for animal studies in sterile phosphate buffered saline (PBS; pH 7.4; diluent). ODE 12.5% has been previously shown to elicit optimal experimental outcomes in mice and is well-tolerated [6].

### 2.3. Animals

All the animal procedures were approved by the Institutional Animal Care and Use Committee at the University of Nebraska Medical Center and were in accordance with the NIH guidelines for the use of rodents. C57BL/6 mice were purchased from The Jackson Laboratory (Bar Harbor, ME). Male mice, between 7–10 weeks, were used for all studies. All mice had *ad libitum* access to standard rodent chow and filtered water through the course of the studies.

### 2.4. Animal exposure model

An established intranasal inhalation repetitive exposure animal model was utilized whereby mice were lightly sedated under isoflurane and received treatment with either 50 μL of sterile saline (PBS) or 12.5% ODE daily for 3 weeks [6,12,22,23], which is denoted as “repetitive ODE treatments.” For recovery period time point experiments, mice were treated daily for 3 weeks and allowed to recover for 1, 2, 3, or 4 weeks without treatments, which is denoted as “post-injury recovery period.” At specified time points, animals were euthanized for experimental endpoint quantification. The protein concentration of each ODE treatment was 149 ug ± SD of 6 μg as measured by spectrophotometry (NanoDrop Technologies, Wilmington, DE). No mice exhibited respiratory distress, signs of stress, or weight loss throughout the treatment period.

### 2.5. Bronchoalveolar lavage fluid cell analysis

Bronchoalveolar lavage fluid (BALF) was accumulated using 3 × 1 mL PBS. Total cell numbers from the three pooled lavages were enumerated and differential cell counts were determined from cytospin-prepared slides (cytopro cytocentrifuge, ELITech Group, Logan, UT) stained with DiffQuick (Siemens, Newark, DE). From cell-free supernate of the first lavage fraction, amphiregulin was quantitated by ELISA (R&D Systems, Minneapolis, MN).

### 2.6. Histopathology

Following lung lavage, whole lungs were excised and slowly inflated (20 cm H_2_O pressure) with 10% formalin (Sigma) for 24 hours to preserve pulmonary architecture as previously described [6]. Fixed lungs were processed, embedded in paraffin, and entire lung sections were cut (4–5 μm) and stained with hematoxylin and eosin (H&E). Each slide was entirely reviewed at scanning magnifications (2X, 4X, and 10X objectives; Nikon Eclipse Model E600 microscope, Nikon, Tokyo, Japan) and semi-quantitatively assessed for the degree and distribution of lung inflammation by a pathologist (W.W.W.), blinded to the treatment conditions, utilizing a previously published scoring system [6]. This scoring system evaluates the spectrum of inflammatory changes for: 1) alveolar compartment inflammation, 2) bronchiolar compartment inflammation, and 3) intrapulmonary cellular aggregates. Each parameter was independently assigned a value from 0 to 3, and the greater the score, the greater the inflammatory changes in the lung.

### 2.7. Flow cytometry phenotyping of whole lung cells

Cells were isolated from whole lungs as previously described [12,22,23]. Briefly, following euthanasia and lung lavage, the right ventricle was infused with 10 mL sterile PBS to remove blood from the pulmonary vasculature. Next, lungs were harvested and subjected to an automated dissociation procedure using a gentleMACS dissociator instrument according to manufacturer instructions (Miltenyi Biotech, Auburn, CA) in a solution containing collagenase type I (324 U/mL; Fisher, Pittsburgh, PA), bovine DNase (75 U/mL), porcine heparin (25 U/mL) and PBS with Ca^2+^ and Mg^2+^ (pH 7.4). The resulting suspension was passed through a nylon mesh (40 μM; Thermo Fisher Scientific, Waltham, MA) to remove any large fragments. The red blood cells were subsequently lysed using a 0.84% (w/v) ammonium chloride treatment (5 min at 4°C), and after centrifugation at 425 × *g*, the remaining cells were re-suspended in PBS and final cell counts obtained using a hemocytometer. Viability of the final cell preparations was assessed by trypan blue exclusion. A LIVE/DEAD Fixable Violet Dead Cell Stain kit (Life Technologies, Carlsbad CA) was also used to assess cell viabilities. More than 99% of gated macrophages and lymphocytes were viable, with no differences noted among the saline, repetitive ODE, and post-ODE recovery-treatment groups (data not shown). Whole lung cells from each animal were incubated with anti-CD16/32 (F_c_ Block, BD Biosciences, San Jose, CA) to minimize non-specific antibody staining, and then stained with monoclonal antibodies (mAb) directed against Ly-6G, CD11c, CD11b, CD3, CD4, CD8, CD45R/B220. Parallel cell preparations were treated with appropriate isotype controls (BD Biosciences). Cytometer compensation was performed with antibody capture beads (BD Biosciences) stained separately with individual mAbs used in test samples.

The gating strategy for Ly6G^+^neutrophils, CD11c^hi^CD11b^lo^ alveolar macrophages, CD11c^hi^CD11b^hi^ exudative macrophages, CD3^+^CD4^+^ and CD3^+^CD8^+^ T lymphocytes, and CD3^−^CD45R/B220^+^ B lymphocytes are as previously published [12,22,23]. All populations were gated by characteristic forward and side-scatter properties and antibody-specific staining fluorescence intensity using a FACSAria cell sorter system and associated software (BD Biosciences). Briefly, initial gating on CD45^+^ lung leukocytes excluded debris, and the percentage of respective populations (i.e., macrophage, lymphocyte, neutrophil) was determined from CD45^+^ leukocytes. This cytometric percentage (determined by flow cytometry) is multiplied by the original hemocytometer count of total cells recovered for each animal. In each case, a minimum of 50,000 CD45^+^ events/mouse sample was acquired for analysis.

### 2.8. Apoptosis Immunohistochemistry Assay

Apoptotic lung cells were determined by TUNEL staining of tissue sections using the ApopTag Peroxidase In Situ Apoptosis Detection Kit (Millipore, Billerica, MA) according to manufacturer’s instructions. Slides were scanned (Ventana Roche Coreo Scanner AU, Tucson, AZ) and photographs were taken of lung cellular aggregates by Ventana Roche Image viewer computer software. Image-Pro Analyzer software determined the percent of apoptotic cells in the 40× cellular aggregates. A minimum of 32 images from 4 mice per treatment group were analyzed.

### 2.9. Serum

Whole blood was collected from mice at the time of euthanasia from the axillary artery. Blood (400 μL) was placed in BD Microtainer Tubes (Becton, Dickinson and Company, Franklin Lakes, NJ) and centrifuged for 2 minutes at 6000 × g and supernatant sample collected. Serum IgG and IgE were quantified according to manufacturer’s instruction using a Quantikine enzyme-linked immunosorbent assay kit (Affymetrix eBioscience, Santa Clara, CA) with sensitivities of 1.56 ng/mL and 4 ng/mL, respectively.

### 2.10. Statistical methods

Data are presented as the mean ± standard error of mean (SEM). To detect significant changes between groups, a one-way analysis of variance (ANOVA) was utilized and a post hoc test (Tukey/LSD) or nonparametric Mann-Whitney test was performed to account for multiple comparisons if the *p* value was < 0.05. All statistical analysis were performed using SPSS software (SPSS, Chicago, IL, USA) and statistical significance accepted at *p* < 0.05.

## 3. Results

### 3.1. Airway cellular influx in BALF after repetitive ODE treatments and following the 1–4 week post-injury recovery periods

**Figure 1:**
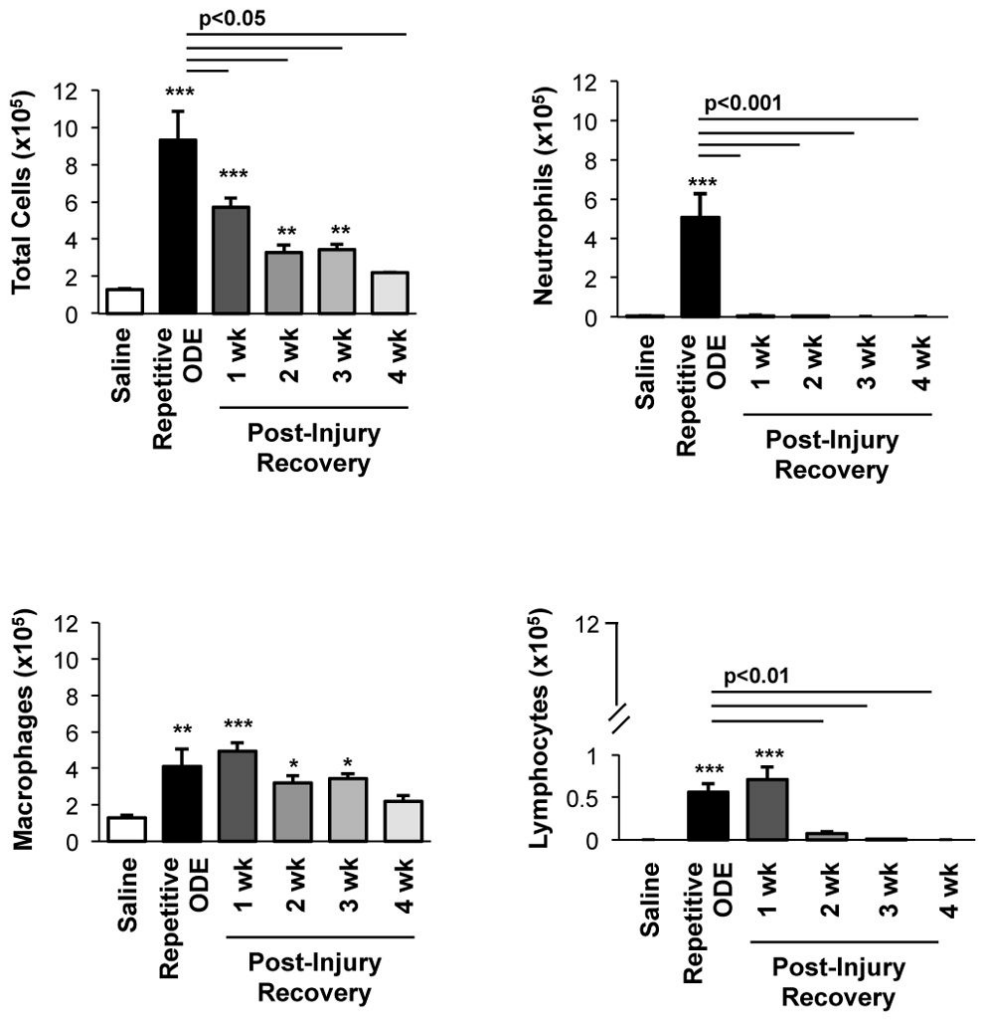
Airway cellular influx in bronchoalveolar lavage fluid after repetitive ODE treatments and subsequent 1–4 week post-injury recovery time periods C57BL/6 mice were intranasally treated with saline or ODE daily for 3 weeks (repetitive ODE exposure) or treated with ODE daily for 3 weeks followed by no treatments for 1, 2, 3 or 4 weeks (recovery time periods). Bar graphs show mean with standard error bars of BALF counts of total cells, neutrophils, macrophages, and lymphocytes (N=8–9 mice/treatment group from 2 independent experiments). Statistically significance (*p<0.05, **p<0.01, ***p<0.001) versus saline. Significant differences between repetitive ODE and post-recovery time points as indicated by line.

It has been established that repetitive ODE treatment induces the influx of neutrophils, macrophages, and lymphocytes, and that airway macrophages and lymphocytes remain increased at one week following final ODE treatment [12]. In the present study, we investigated the temporal course for the resolution of repetitive ODE-induced inflammatory cell influx (Figure 1). BALF neutrophils were cleared by 1 week post-exposure (p<0.001). However, macrophage counts remained elevated for 3 weeks following final ODE treatment. ODE-induced lymphocyte counts remained increased at 1 week post-injury, and remained detectable, but notably decreased, up to 3 weeks after final ODE treatment. Eosinophils were not detected in any of the treatment conditions.

### 3.2. Recovery from repetitive ODE exposure is marked by persistence of cellular aggregates for up to 4 weeks in the lung tissue

**Figure 2:**
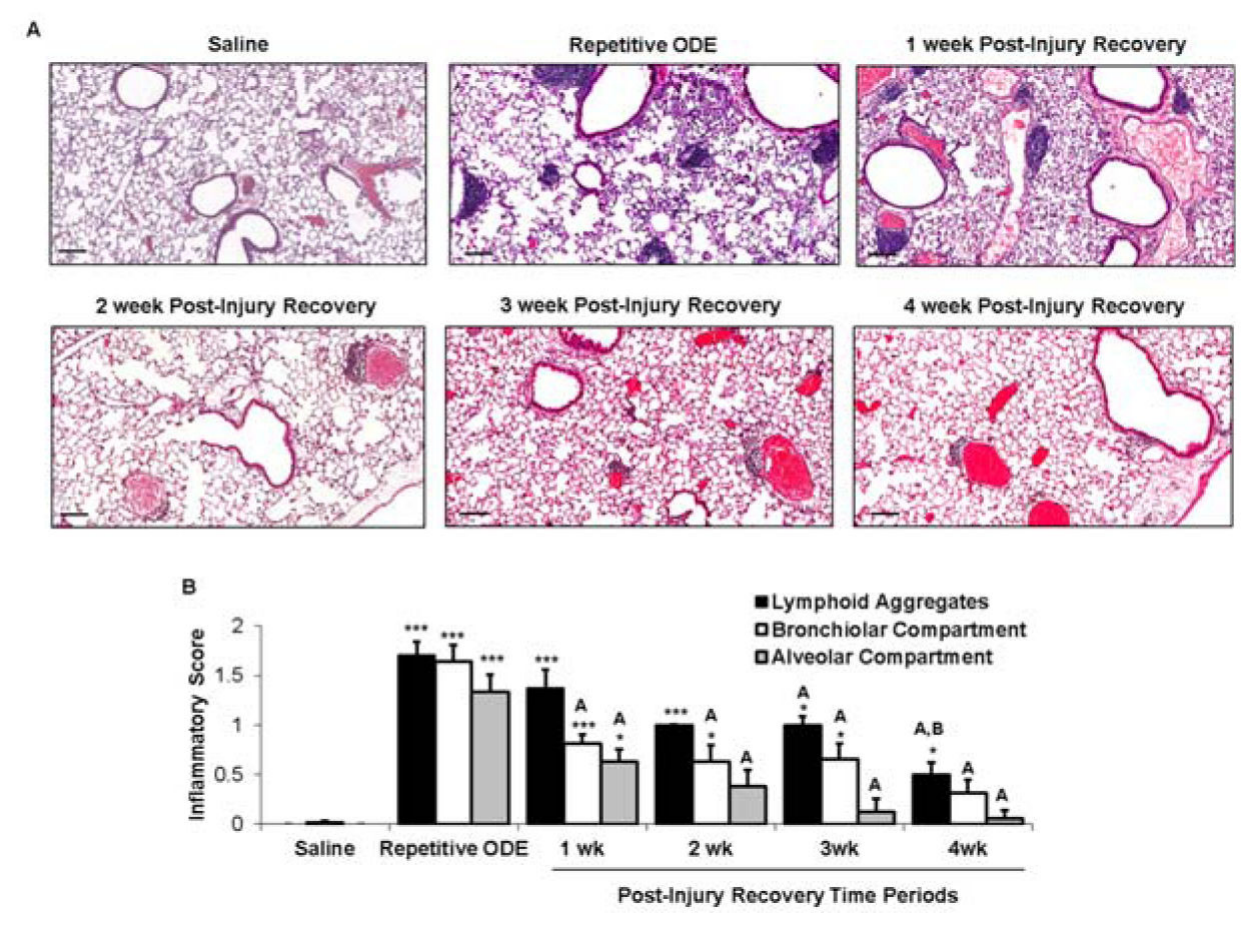
Recovery from repetitive ODE exposure is marked by persistent cellular aggregates for up to 4 weeks in the lung tissue C57BL/6 mice were intranasally treated with saline or ODE daily for 3 weeks (repetitive ODE exposure) or treated with ODE daily for 3 weeks followed by no treatments for 1, 2, 3 or 4 weeks (post-injury recovery time periods). A, Representative 4–5-μm thick murine lung section (H&E) stained from each treatment group (10 X magnification) is shown. Line scale is 100 μm. B, Mean semi-quantitative distribution of inflammatory scores of lung cellular aggregates, bronchiolar inflammation, and alveolar inflammation in mice (N=5 mice/treatment group). Error bars represent SEM. Statistical significance (*p<0.05, ***p<0.001) versus saline. Statistical significance also denoted by A: p<0.05 vs. repetitive ODE treatment; B: p<0.05 vs. 1 week recovery time point.

Repetitive ODE treatment induces lung inflammation marked by the recruitment of inflammatory cells into the bronchiolar and alveolar compartments as well as development of cellular aggregates [6]. Here, we sought to define the normative time course of restoration to pre-ODE-induced lung pathology. Repetitive ODE treatment induced the characteristic increase in inflammatory cells and development of cellular aggregates (Figure 2A), and moreover, by microscopic review, these pathologic findings remained detectable up to 4 weeks following final ODE treatment. The range of ODE-induced histopathologic change was semi-quantitatively assessed in a blinded manner by a pathologist (Figure 2B). As compared to saline, the degree and distribution of lung alveolar inflammation remained significantly increased until 2 weeks post-injury and bronchiolar inflammation remained significantly increased until 4 weeks. As compared to repetitive ODE treatment, there was significant evidence of a decrease in both alveolar and bronchiolar inflammation at 1 week post-injury recovery period. ODE-induced cellular aggregates demonstrated prolonged persistence. At 4 weeks post-injury, the size and distribution of ODE-induced cellular aggregates remained significantly increased as compared to saline treatment.

### 3.3. Clearance of repetitive ODE exposure-induced lung neutrophils, alveolar macrophages, and exudative macrophages is time-dependent

**Figure 3:**
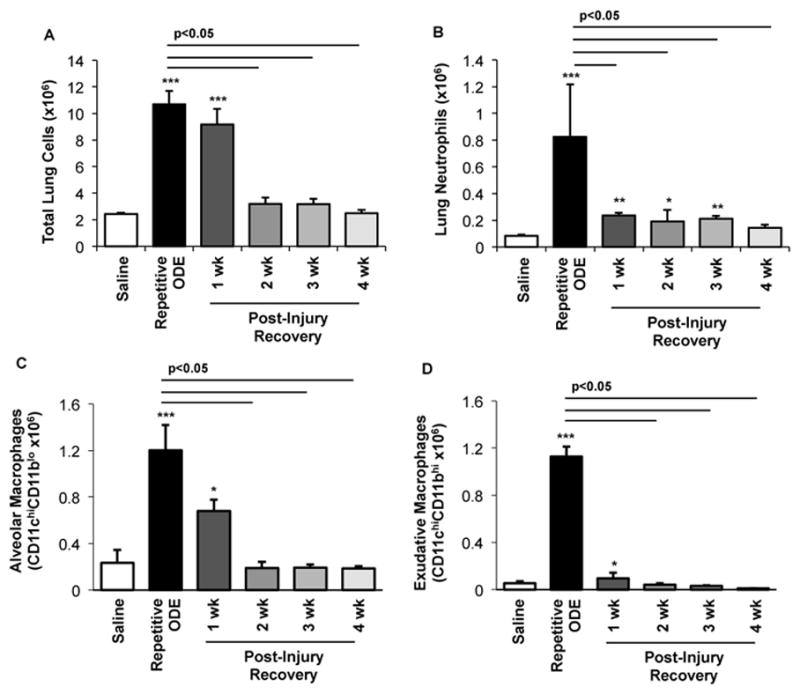
Clearance of repetitive ODE exposure-induced lung neutrophils, alveolar macrophages, and exudative macrophages is time-dependent Mice were intranasally treated with saline or ODE daily for 3 weeks (repetitive ODE exposure) or treated daily with ODE for 3 weeks followed by no treatment for 1, 2, 3, or 4 weeks (post-injury recovery time periods) whereupon mice were euthanized, lavage fluid removed, and lung cells dissociated. A, Mean total lung cells as determined by hemocytometer. B, Total neutrophils (Ly6G^+^), C, Alveolar macrophages (CD11c^hi^CD11b^lo^), and D, Exudative macrophages (CD11c^hi^ CD11b^hi^). Number of lung neutrophils and macrophages were calculated by multiplying the percentage of cells in respective gate (% of CD45^+^ cells, as analyzed by FACs) multiplied by total lung cells for each mouse. Bar graphs depict means with standard error bars. N=4–5 mice/group. Statistically significance (*p<0.05, **p<0.01,***p<0.001) versus saline. Line denotes significant difference (p<0.05) between repetitive ODE and a post-recovery timepoint.

To further delineate the post-injury inflammatory cellular effect observed in the lung tissue, cells were dissociated from whole lung and analyzed as described in the Methods section following repetitive ODE treatment and post-injury recovery time periods. Repetitive ODE treatment resulted in increased total whole lung cell influx that persisted following the 1-week post-injury as compared to saline treatment (Figure 3A). Lung neutrophils were significantly decreased by 1 week post-injury, but remained elevated as compared to saline control until 4 weeks post-injury recovery time period (Figure 3B). Alveolar CD11c^+^CD11b^lo^ macrophages and exudative CD11c^+^CD11b^hi^ macrophages induced by repetitive ODE exposure remained increased at 1 week post-injury as compared to saline, but were significantly decreased as compared to repetitive exposure (Figure 3C–D).

### 3.4. Lymphocytes, particularly CD4^+^ and CD8^+^ T cells, demonstrated prolonged persistence in the lung following final ODE exposure

**Figure 4:**
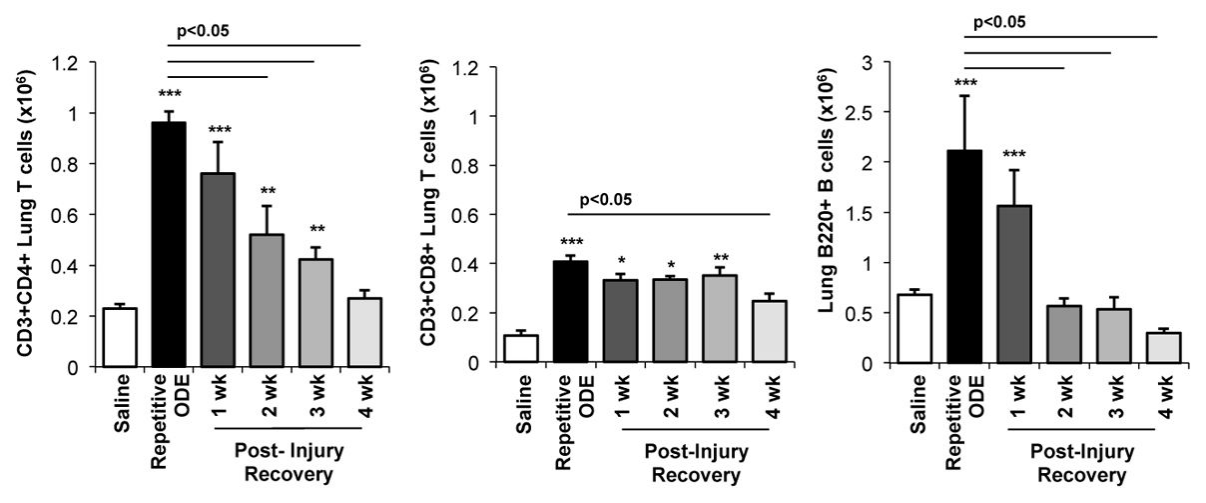
Lymphocytes, particularly CD4^+^ and CD8^+^ T cells, demonstrated prolonged persistence in the lung following removal of ODE Mice were intranasally treated with saline or ODE daily for 3 weeks (repetitive ODE exposure) or treated daily with ODE for 3 weeks followed by no treatment for 1, 2, 3, or 4 weeks (post-injury time periods) whereupon mice were euthanized, lavage fluid removed, and lung cells dissociated. Lymphocytes were identified by CD45 positivity and characteristic FSC and SSC properties of lymphocytes followed by staining for CD3, CD4, CD8, and B220. Total numbers of each lymphocyte population were determined by multiplying the frequency of CD3^+^CD4^+^ cells, CD3^+^CD8^+^ cells, or CD3^−^B220^+^ cells (among the CD45^+^ leukocytes) by the total lung cell number for each mouse. Bar graph depicts mean with standard error bars of N=4–5 mice/treatment group. Statistical significance (*p<0.05, **p<0.01, ***p<0.001) versus saline. Line denotes significant difference (p<0.05) between repetitive ODE and a post-recovery time point.

To further understand the persistent lymphoid aggregate response, the infiltrating lung lymphocyte phenotypes were investigated by flow cytometry. Consistent with prior reports [23], repetitive ODE treatments resulted in increased levels of CD3^+^CD4^+^ and CD3^+^CD8^+^ T cells as well as increased B cells (B220^+^) (Figure 4). In the post-recovery time conditions, we found a gradual decrease in the number of CD3^+^CD4^+^ cells out to 4 weeks following final ODE treatment. The number of CD3^+^CD8^+^ T cells remained increased out to 3 weeks. In comparison, clearance of B cells predominately occurred by 2 weeks post-repetitive ODE-induced lung injury.

### 3.5. Apoptotic cells within cellular aggregates diminished over time following final repetitive ODE exposure

**Figure 5:**
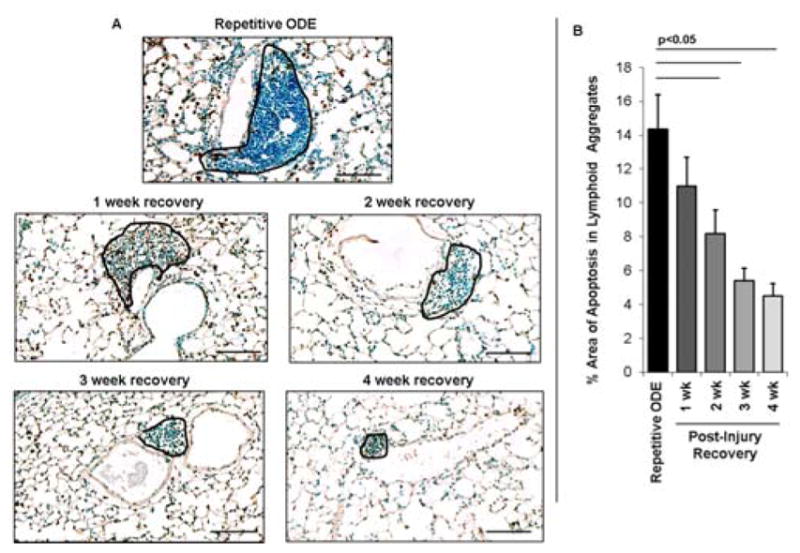
Apoptotic cells within cellular aggregates diminish over time following final repetitive ODE exposure Mice were intranasally treated with ODE for 3 weeks or treated repetitively with ODE followed by no treatment for 1, 2, 3, or 4 weeks (post-injury recovery time period). **Panel A,** representative 4–5-μm thick section stained using Apoptag for apoptotic cells of a mouse from each treatment group (20 X magnification) with cellular aggregates outlined in black. Line scale is 100μm. **Panel B,** Bar graphs depicts mean percentage area of positive apoptotic events within cellular aggregates with SE bars. A minimum of 32 images from 4 mice per group were analyzed using Image-Pro Analyzer software. Line denotes significant difference (p<0.05) between repetitive ODE and a post-injury recovery time periods.

The pattern of lung cell apoptosis events in post-injury tissue homeostasis was investigated by TUNEL assay. By microscopic review, lung apoptotic cells were greatest within the cellular aggregates with repetitive ODE exposure, and there was evidence of diminishing apoptotic events within the cellular aggregates in recovery time periods (Figure 5A). To quantitate these observed differences in post-injury recovery time periods, the area of cellular aggregates from 8 images per mouse (N=4 mice) per treatment group were assessed for percent positive staining cells per area. As shown in Figure 5B, there was active apoptosis with repetitive exposure (~14%) within the cellular aggregates, and these events were reduced to approximately 5% of the cellular aggregates at 4 weeks recovery.

### 3.6. Levels of serum immunoglobulins persisted and BALF amphiregulin levels increased over time following repetitive ODE exposure

**Figure 6:**
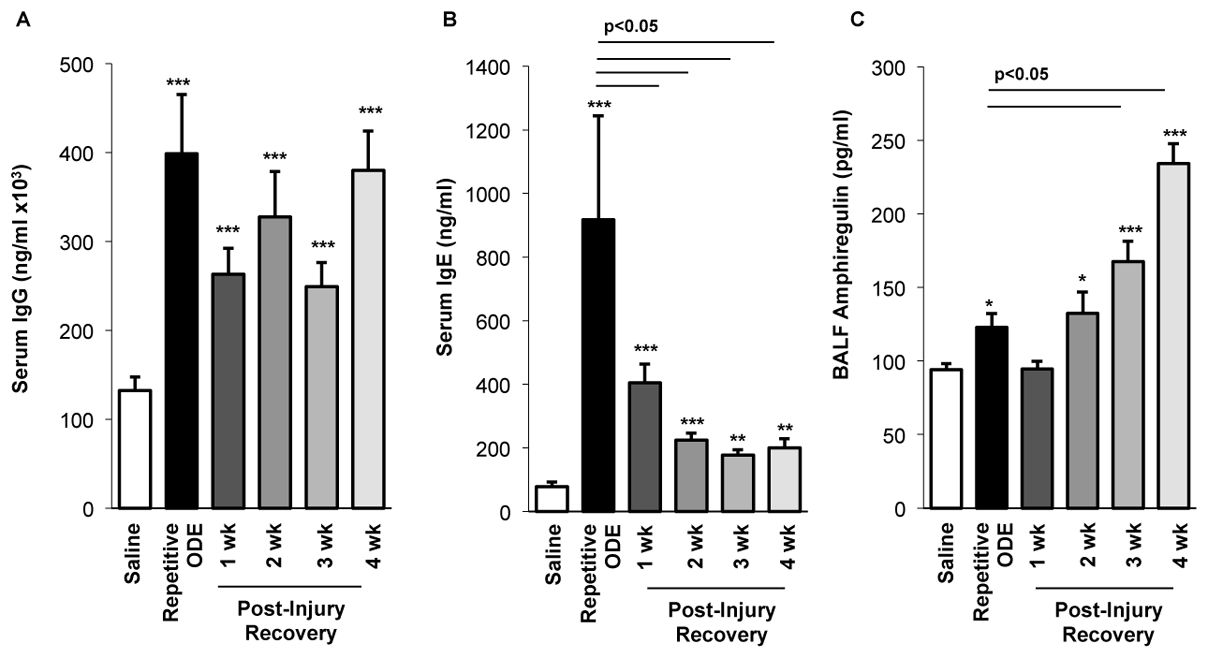
Serum immunoglobulin and bronchoalveolar lavage fluid amphiregulin levels over time following repetitive ODE exposure Mice were intranasally treated with ODE for 3 weeks or treated daily for 3 weeks with ODE followed by no treatment for 1–4 weeks (post-injury recovery time period). A, Serum IgG and (B) IgE levels were increased following repetitive ODE exposure and remained elevated as compared to saline control for up to 4 weeks cessation of ODE exposure. C, Amphiregulin levels in BALF increased over time following cessation of repetitive ODE exposure. Bar graphs depict mean with standard error bars of N=4–9 mice/treatment group. Statistically significance (*p<0.05, **p<0.01, ***p<0.001) versus saline. Line denotes significant difference (p<0.05) between repetitive ODE and a post-injury recovery time point.

To determine whether a systemic adaptive immune response resulting from repetitive ODE exposure was present and/or persisted following cessation of ODE exposure, serum levels of murine IgG and IgE were investigated. For the first time, we demonstrated that repetitive ODE exposure increases both serum IgG and IgE levels, and IgG levels remain elevated for 4 following cessation of ODE treatment (Figure 6A–B). Whereas serum IgE levels also remain increased as compared to saline for up to 4 week recovery time, there was a significant decrease in IgE levels following one week post-injury as compared to repetitive exposure condition (Figure 6B).

Amphiregulin, produced by a number of cells including type 2 innate lymphoid cells, T regulatory cells, macrophage and lung epithelial cells, has been implicated in lung repair [25–27]. Following repetitive ODE exposure we detected a small, but significant increase in amphiregulin in the BALF (Figure 6C). Interestingly, amphiregulin levels continued to rise over the post-injury recovery time points, with maximal levels detected at 4 weeks post cessation of ODE treatment (Figure 6C).

## 4. Discussion

In this study, we defined the post-injury lung homeostasis response following repetitive ODE exposure over a four-week recovery period. Our study characterized ODE-mediated neutrophil, macrophage and lymphocytic recruitment and regression in the airways and the lung parenchyma. Furthermore, we demonstrated prolonged persistence of neutrophils and lymphocytes and the corresponding resolution of cellular aggregates following repetitive exposure to ODE. Apoptotic events appear important in the early post-recovery phases of the normative resolution response. Studies demonstrated that a systemic adaptive immune response was induced by repetitive ODE exposure, which was evident by increased serum IgG and IgE levels that persisted for up to 4 weeks post cessation of ODE. The pro-repair/resolving mediator, amphiregulin, was also induced in BALF following repetitive ODE exposures, but was found at greatest levels at 3 and 4 weeks post-injury. These studies provide time-dependent insight into the normative repair and resolution response following ODE exposure in an animal model.

Neutrophils have been characterized in many studies for their ability to mount immunity to various pathogens and subsequently aid in the establishment of tissue homeostasis following injury [28,29]. In the present study, neutrophil counts return to baseline in the airways within the first week following ODE exposure; however, neutrophils remained present in the lung parenchyma for up to 3 weeks following cessation of exposure. Neutrophils are short-lived, and normally undergo apoptosis once they have fulfilled their role in responding to inflammatory insult [21,30]. Persistence of neutrophils is commonly thought to lead to chronic inflammation and host tissue damage in lung diseases such as neutrophil-variant asthma and COPD [31]. Alternatively, there is also evidence that neutrophils can change from a pro-inflammatory to an anti-inflammatory phenotype in response to the lung microenvironment and act to enhance the resolution of inflammation [28,32]. Thus, it is possible that the prolonged presence of neutrophils in the lung tissue, but not the airways, following cessation of ODE exposure might fit into this new paradigm. Future studies could focus on understanding the phenotypic specificity of the post-inflammatory lung neutrophil function as well as location and distribution within the lung after repetitive ODE exposure. However, others have recently shown that an amplification of neutrophil apoptosis facilitated wound healing in an endotoxin-induced experimental model of acute respiratory distress syndrome [30], which would suggest that enhancing neutrophil apoptosis is beneficial.

Macrophages are well-described regulatory immune cells [33,34]. Initially, the macrophage serves to trap pathogens and establish an appropriate degree of inflammation. In the recovery period, lung macrophages are indispensable to lung recovery [22,35]. In the present study, macrophages composed the greatest portion of the BALF for out to 4 weeks of recovery. In contrast, the activated or exudative CD11c^+^CD11b^+^ lung macrophage and CD11c^+^CD11b^lo^ alveolar macrophage populations in the whole lung were only increased at the first week following cessation of ODE treatment. Exudative or activated CD11c^+^ lung macrophages are a common feature of inflammatory responses [36], and in organic dust extract focused studies, the CD11c^+^CD11b^+^ lung macrophage with M1 features has been demonstrated [22,37]. Here, levels of these macrophages diminished by 2 weeks post-injury (Figure 3). Predominately through their phagocytic capabilities, macrophages are important in resolution of inflammatory responses to efficiently clear apoptotic cells, a process called efferocytosis [21,38]. Our data demonstrate that apoptotic events were increased early in the recovery period, which, to some degree, follows the pattern of macrophage localization in the lung parenchyma. We have previously reported that depletion of lung macrophages results in accumulation of neutrophils following repetitive ODE exposure [22]. Thus, our finding of relative rapid resolution of macrophages in the lung tissue might also suggest a dysregulated repair response. This suggests that strategies aimed at enhancing macrophage function and/or numbers to improve ODE-induced lung injury might be necessary. Moreover, further delineation of the macrophage phenotype and function might be warranted, particularly because it was demonstrated by others that classically activated M1 macrophages produce high concentrations of amphiregulin to control lipopolysaccharide-induced acute lung injury [39].

Lymphocytes, cellular aggregates and apoptotic events within cellular aggregates were also characterized. Lavage lymphocyte levels decreased at a rate coincidental to lymphoid aggregate resolution. In prior work, the cellular aggregates were found to be composed of B cells, CD3^+^ T cells, and Mac3^+^ macrophages [6]. It is not known at this time whether the ODE-induced lung cellular aggregates develop high endothelial venules, a feature of inducible bronchus-associated lymphoid tissue [40,41]. However, we previously demonstrated in independent phenotyping investigations that the CD3^+^ T cells were present in the lungs with a strong CD4^+^ T cell population skewed toward a Th1/Th17-characterized microenvironment [23]. However, in these current studies, Th1/Th2/Th17/Treg cytokines were not detected in the lung homogenates or BALF in the post-injury time points (data not shown). It remains possible that we were not able to detect local production of Th1/Th17 cytokines, which could be further explored in the future by isolating various candidate lung cells and subsequently applying ex vivo culture stimulation conditions. Alternatively, it is possible that the regulatory FoxP3^+^ T-cell subset, known to produce IL-10, plays a local role in resolution and repair [42–44]. We speculate that the cellular aggregates are likely resolved as antigenic stimulation is removed from the lung.

Less is known about the B cell response following organic dust exposure, and in this study, we found that systemic IgG and IgE levels were increased following repetitive ODE exposure; moreover, levels remained elevated for up to 4 weeks following cessation of exposure. Class-specific IgG and IgE immunoglobulins have been associated with Farmer’s lung disease [45,46]. Given the complexity of the organic dust exposure, the specific antigen(s) eliciting this immunoglobulin response are currently not known. It is possible that a spectrum of antibodies may be important in complexing ODE components to effectively attract and aggregate innate and adaptive immune cells for optimal clearance and resolution of injury. To our knowledge, this is the first animal model to demonstrate an increased and prolonged immunoglobulin response to complex organic dust extract exposures, which could lead to novel strategies focused on B-cell biology.

Amphiregulin is an endogenous pro-repair mediator that has been increasingly implicated in the resolution and repair of asthmatic lung disease [24,25] and nonallergic chronic lung disease [47,48]. In this present study, amphiregulin was slightly increased with repetitive ODE exposures, but interestingly, levels of amphiregulin further increased in the post-injury recovery time periods following cessation of ODE treatment. The observation of increasing levels of amphiregulin would support studies focused on innate lymphoid cells, Tregs, epithelial cells, and/or macrophages as others have demonstrated these cells as potential sources of amphiregulin [24,26,27]. Furthermore, amphiregulin could be potentially exploited in future studies aimed at enhancing repair and injury induced by organic dusts.

There are other limitations of this study. The ODE utilized is a sterile, aqueous extract, and this process could eliminate other potential inflammatory agents such as whole bacteria and fungal spores from being investigated. Next, we utilized an intranasal inhalation delivery method, which is also being utilized by others in this field [37]. However, other animal exposure models that have been utilized include hanging murine cages in swine confinement facilities [13], intratracheal instillation [49], and aerosolized delivery systems [50].

In conclusion, agricultural dust exposures cause significant morbidity and loss of work hours and productivity in individuals regularly exposed. Here, we determined the timing of the normative recovery phase following ODE and show in detail the timing and kinetics of airway immune cell influx and regress. This ODE exposure-recovery model can be used in future studies where determining the efficacy of resolution-promoting targets may be tested to determine potential translational therapies for agriculture workers exposed to organic dust.
